# The Effects of Bariatric Surgery on Pharmacokinetics of Drugs: a Review of Current Evidence

**DOI:** 10.1007/s13668-023-00498-5

**Published:** 2023-10-20

**Authors:** Sofia K. Konstantinidou, Georgia Argyrakopoulou, Maria Dalamaga, Alexander Kokkinos

**Affiliations:** 1 First Department of Propaedeutic Internal Medicine, School of Medicine, Laiko General Hospital, National and Kapodistrian University of Athens, Athens, Greece; 2 Diabetes and Obesity Unit, Athens Medical Center, 15125 Athens, Greece; 3 Department of Biological Chemistry, School of Medicine, National and Kapodistrian University of Athens, 11527 Athens, Greece

**Keywords:** Bariatric surgery, Drugs, Absorption, Obesity, Pharmacokinetics

## Abstract

**Purpose of Review:**

Obesity constitutes a major public health concern and has been recognized as an epidemic. To date, bariatric surgery remains the most effective way for substantial long-lasting weight loss in severe obesity. The purpose of this review is to summarize how the pharmacokinetics of drugs are affected by the most common types of bariatric surgery, i.e., Roux-en-Y gastric bypass (RYGB) and sleeve gastrectomy (SG).

**Recent Findings:**

Limited data are available regarding the changes in pharmacokinetics of drugs after bariatric surgery. The lack of existing guidelines may lead patients to experience drug toxicity or therapeutic undertreatment. Pharmacokinetic parameters that need to be taken into consideration postoperatively include gastric motility, gastric volume, pH, surface area, bile secretions, carrier proteins, and first-pass metabolism. For drugs with a narrow therapeutic index, other factors need to be monitored closely, including plasma drug levels, patients’ clinical outcomes, and laboratory markers. Patients should be followed up frequently and treated in accordance with their response to the drug therapy.

**Summary:**

Bariatric surgery may affect the pharmacokinetics of various drugs, due to the resultant anatomical changes and the substantial weight loss. Therefore, there is a need to identify those potential changes and adjust patients’ medication doses in order to achieve higher efficacy and avoid toxicity.

## Introduction

Obesity constitutes a major public health concern and has been recognized as an epidemic. It is defined as excessive fat accumulation that may impair health, considered a multisystem condition with various implications for patients, as well as the society as a whole [1]. Its prevalence has increased dramatically during the last decades. In 2016, more than 1.9 billion adults were overweight while over 650 million were obese [1]. Moreover, in 2020, 39 million children under the age of 5 were overweight or obese [1]. Obesity is associated with multiple comorbidities, such as type 2 diabetes mellitus (T2DM), hypertension, cardiovascular disease (CVD), hyperlipidemia, cancer, and depression [2]. Pharmacological therapy is usually needed for the treatment of both obesity and its accompanying diseases, while oftentimes, polypharmacy is deemed necessary.

Apart from the health, social, and economic burden, obesity also poses a risk to physicians’ medical decisions. The reason is that patients’ drug doses may need to be readjusted in order to avoid toxicity and achieve efficacy, as their pharmacokinetics and physiological effects may be altered due to obesity. Therefore, optimizing pharmacotherapy is of paramount importance in the treatment of patients with obesity.

Obesity is managed with lifestyle modifications (diet and exercise), medications, or bariatric surgery, which is indicated for patients with body mass index (BMI) ≥ 40 kg/m^2^ or ΒΜΙ ≥ 35 kg/m^2^ with significant comorbidities, i.e., T2DM, hypertension, sleep apnea, or benign intracranial hypertension [3]. To date, bariatric surgery remains the most effective way for substantial long-lasting weight loss in patients with severe obesity [4•, 5•, 6].

Continuous data have shed light into the complicated mechanism of weight loss through bariatric surgery including the upstream/downstream regulation of gut hormones, which eventually leads to enhanced satiety and, thereby, reduced food consumption. However, bariatric procedures may be associated with restrictive and malabsorptive effects, i.e., reducing the amount of food that can physically be consumed [7].

All types of malabsorptive procedures lead to decreased absorption surface in the small intestine and decreased exposure to bile acids and enterohepatic circulation, whereas restrictive operations additionally lead to increased stomach pH and decreased contact time with digestive enzymes [8•]. There are several types of techniques used in bariatric operations, including the adjustable gastric band, sleeve gastrectomy (SG), Roux-en-Y gastric bypass (RYGB), biliopancreatic diversion (BPD), or biliopancreatic diversion with duodenal switch (BPD-DS) [3]. Statistical data from the International Federation for the Surgery of Obesity and Metabolic Disorders (IFSO) worldwide survey showed that in 2016, the most commonly performed type of bariatric operation was SG (54%), an operation leading to a degree of restriction, and the second most common was RYGB (30%), which has both a restrictive and a malabsorptive element [9]. The first type limits only food intake, whereas the second type additionally limits nutrient absorption [5•]. Drug absorption and bioavailability can be affected postoperatively, creating the need for change in drug dosage and administration [10].

*Pharmacokinetics*, i.e., the movement of a drug through the body compartments, is defined as the study of the time course of the drug’s absorption, distribution, metabolism, and excretion, in order to account for the safe and effective therapeutic management of the patients’ medication and therapeutic management [11]. The goal is to enhance efficacy and decrease toxicity. On the other hand, *pharmacodynamics*, i.e., the body’s biological response to drugs, describes the relationship between drug concentration at the site of action and the resulting biological effect [11].

To date, very few studies have focused on how bariatric surgery procedures may affect drugs’ pharmacokinetics. The aim of this review is to explore how the most common types of bariatric procedures, i.e., RYGB and SG, could have an impact pharmacokinetics and to discuss the pharmacokinetic changes of commonly used drugs after bariatric surgery.

## Effects of Obesity on Drug Pharmacokinetics

Obesity itself may affect the pharmacokinetics of some drugs [4•]. Obesity is accompanied by low-grade chronic inflammation, leading to the aberrant secretion of cytokines, chemokines, and adipokines, which may reduce the amount of cytochrome P450 (CYP) enzymes [4•]. People with obesity often suffer from non-alcoholic fatty liver disease (NAFLD) and non-alcoholic steatohepatitis (NASH), which are also known to change the activity of CYP enzymes [4•].

Drug distribution and elimination may be affected by the pathophysiological changes of patients with obesity. Clinicians should take into consideration which “weight type” to use in order to titrate the patient’s dosage, i.e., total body weight (TBW), ideal body weight (IBW), or lean body mass (LBM) [12]. Drug recommendations are typically made with the assumption that pharmacokinetics are weight proportional and thus are calculated based on TBW [12]. Drug’s volume of distribution (*V*_d_), clearance, and protein binding may be affected in patients with obesity [12]. Therefore, drugs with high lipophilicity may have markedly increased *V*_d_, whereas drugs with low lipophilicity may not have any changes in *V*_d_ [12]. Before considering alterations in drug dosing, factors linked to the pharmacokinetics and pharmacodynamics should be considered. Therapeutic drug monitoring is encouraged for drugs with a narrow therapeutic index (e.g., digoxin, aminoglycosides, cyclosporin, carbamazepine, lithium, phenytoin, rifampicin, theophylline, warfarin) and for those that do not have a specific biomarker associated with their efficacy [4•].

## Effects of Bariatric Surgery on Drug Pharmacokinetics

**Fig. 1:**
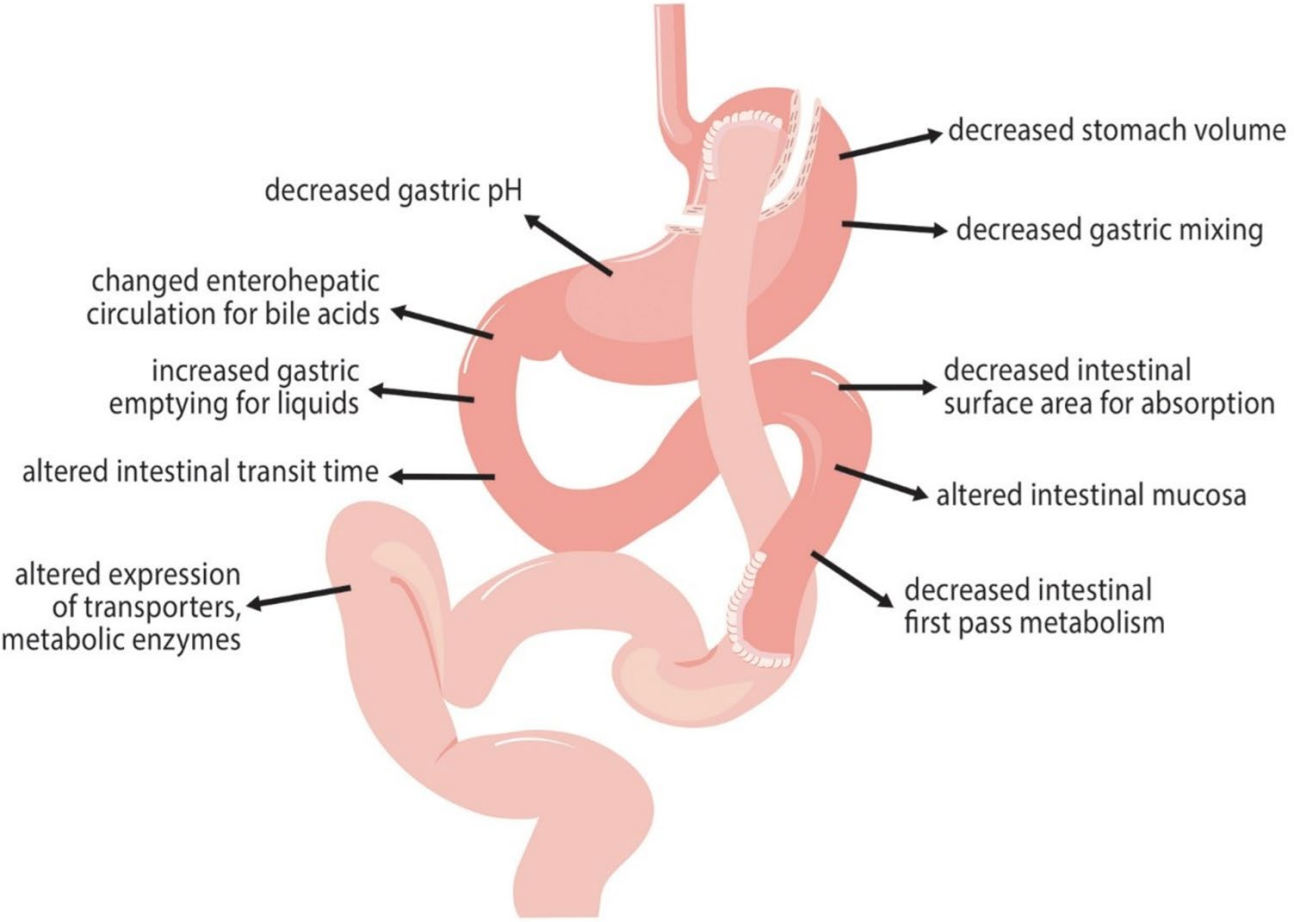
Main factors influencing the absorption of drugs after RYGB

Following bariatric surgery and weight loss, it is important to consider the potential changes in drug pharmacokinetics, both in the short and long term and that there is high inter- and intra-patient variability, which may explain drug toxicity or inadequate therapy. The type of surgery also plays an important role, with the RYGB procedure being more likely to affect the absorption of drugs, due to changes in mucosal exposure and decreased bile-salt mixing (Fig. 1) [13]. Drug factors to consider post-surgery include ionization, stability, lipophilicity, and dissolution, whereas patient factors include gastric emptying, pH, and area of mucosal exposure [13]. Moreover, medication absorption changes may be temporary or permanent; thus, medications often need to be reviewed and any modifications closely monitored. Malabsorptive procedures usually cause permanent changes to drug absorption; however, all changes to a patient’s medication regimen after bariatric surgery need to be followed up and adjusted accordingly [7].

Bariatric procedures lead to anatomical and physiological changes, restricting oral bioavailability (F) of drugs. Pharmacokinetic alterations of orally ingested drugs can occur in many ways. After malabsorptive procedures, the expression of metabolic enzymes which are found in the upper small intestine (such as CYP3A4) changes, thus affecting the bioavailability of some drugs [14]. The majority of studies have showed a faster absorption of drugs postoperatively, due to reduced gastric volume and the faster gastric emptying rate, i.e., earlier transfer of the drug in the intestine, therefore leading to faster absorption [4•, 15]. Another factor to take into consideration is the reduced liver size after bariatric surgery, due to substantial weight loss, which may decrease hepatic metabolism and increase bioavailability of drugs, thus affecting pharmacokinetics and pharmacodynamics [16].

Solid oral dosage forms need to disintegrate in the stomach before dissolution happens and absorption takes place in the gastrointestinal (GI) tract. However, post-surgery, the stomach size is reduced, as well as its contractility; thus, drug dissolution may not happen properly for drugs with poor solubility [5•]. Furthermore, gastric pH after surgery is increased, as the acid-producing parietal cells are decreased; thus, the dissolution of pH-dependent drugs may be affected [5•]. Lipophilic drugs may also be inadequately solubilized, as the duodenum and upper part of the small intestine are bypassed; therefore, bile and pancreatic secretions are affected [5•]. RYGB may also change the absorption of drugs that need particular transporters found in the small intestine, e.g., P-glycoprotein, multidrug resistance-associated proteins, and breast cancer resistance protein.

After bariatric surgery, some drugs that may cause GI irritation and delayed healing should be avoided or at least their intake should be minimized, i.e., non-steroidal anti-inflammatory drugs (NSAIDs), oral bisphosphonates, and corticosteroids [17]. Moreover, alterations on the choice of drugs should be made for those that are thought to be inappropriately absorbed. Oral pharmaceutical forms, such as liquids and dissolvable or crushable tablets/capsules, are easier absorbed than solid forms [7, 18, 19]. However, due to the dumping syndrome risk, i.e., rapid gastric emptying, liquid formulations with non-absorbable sugars (e.g., mannitol, sorbitol) should be avoided [5•, 17]. It is also advised to change extended-release to immediate-release formulations. Lastly, for drugs with critical effects on patients’ health, non-oral dosage forms should be considered, i.e., intranasal, sublingual, or subcutaneous formulations [7, 18].

### Absorption

Drug absorption depends on the physicochemical properties of the drug, such as solubility, polarity, lipophilicity, and molecular size. Therefore, different effects are expected depending on the type of the bariatric procedure [4•]. The rate or extent of oral drug absorption does not seem to markedly differ in patients with obesity *vs.* lean individuals. Some examples of drugs studied in bariatric surgery include propranolol, midazolam, and cyclosporine A, which seemed to have unaltered absorption post-surgery [19]. On the other hand, a study by Brocks et al. investigated the absorption of enoxaparin, a low molecular weight heparin (LMWH), which is administered subcutaneously in lean and obese volunteers. Antifactor Xa and antifactor IIa activity levels were measured to determine its pharmacokinetics. The results showed that the rate of the drug absorption was slower in individuals with obesity; however, the extent of absorption was complete in both groups [19]. Nonetheless, an increased absorption rate after bariatric surgery may not always confer significant clinical impact; thus, it is important to take into consideration the affected drug mechanism of action [4•].

An alteration in the gastric pH may affect drug dissolution and solubility. SG seems to affect gastric emptying time and thus drug absorption. On the other hand, RYGB and some other procedures, even though they bypass a large part of the small intestine, do not seem to limit the absorption of drugs. However, the small intestine, which has many metabolizing enzymes, may affect the oral bioavailability of some drugs. Moreover, slow-release formulations may be affected, as the transit time is affected by intestinal motility [4•]. Lipophilic drugs, which depend on bile acids to enhance their solubility, such as phenytoin and thyroxin, that need food or acidic environment to be absorbed should be given extra attention [5•]. Specifically, the secretion of the bile acids is impaired by RYGB, which may lead to the incomplete dissolution and absorption [20]. Thus, it is important to closely monitor patients’ medication use following bariatric surgery to prevent any harmful effects, and if possible, drug levels should be frequently checked via laboratory tests [20].

Paracetamol is a drug that has been studied pre- and post-bariatric surgery, as it is affected by gastric emptying rate. A study showed that 6 months after surgery, the rate and extent of absorption, i.e., the area under the curve (AUC) and the maximum plasma concentration (*C*_max_) of the drug, increased, whereas the time to reach *C*_max_ (*t*_max_) decreased [21]. This is due to the increased gastric emptying after SG and RYGB. Another study found that before surgery, patients with obesity had lower AUC and *C*_max_ compared to lean patients; however, 6 months post-surgery, these parameters were similar in the two groups [21]. The increased rate of absorption after surgery does not necessarily mean increased overall absorption, as in the case of paracetamol systemic exposure remained unchanged [4•].

### Drug Transport

Depending on drug’s solubility and lipophilicity, medications cross the intestinal mucosa by passive diffusion or active transport. Many transport proteins are located in the GI tract, which influences drug absorption, distribution, or elimination. Therefore, some types of bariatric surgery procedures may affect the expression of these proteins [4•]. However, limited data are available on the impact of bariatric operations on intestinal drug transporters. For example, P-gp is important in the absorption and elimination of digoxin. After RYGB, the *C*_max_ and the systemic exposure of the drug were found to be unaffected, whereas its absorption was faster due to the faster gastric emptying [22]. Thus, as the absorption of digoxin is dependent on both GI transit time and P-gp in the small intestine, it is advised to monitor and adjust the dose as required.

### Distribution

Drug distribution depends on the physicochemical properties of the drug, the size of the target tissue, and its physiology. The *V*_d_ represents the volume into which the drug is distributed in the body and can be influenced by increases in adiposity [19]. In particular, the *V*_d_ of lipophilic drugs (e.g., verapamil, diazepam) may increase in patients with obesity, as they penetrate easier into excess tissue stores compared to hydrophilic ones. When the *V*_d_ of a lipophilic drug increases, it means that the drug is distributed more extensively into the body fat tissues, which may decrease the concentration of the drug in the bloodstream. Therefore, to achieve the desired therapeutic effect, the dose of drugs such as verapamil and diazepam may need to be increased to compensate for the larger volume of distribution. Drug dosing based on body mass, e.g., TBW, is often used as a method of dose individualization in patients. Therefore, it is preferable to use TBW, which accounts for the increased adipose mass in order to account for drugs that are highly lipophilic [23]. On the other hand, the *V*_d_ of hydrophilic drugs is expected to decrease, due to the reduced tissue space for the drug to penetrate [23]. This means that these drugs are more concentrated in the bloodstream, and their effects may be more potent than expected; thus, to avoid drug toxicity, the dose of hydrophilic drugs such as metformin, captopril, amoxicillin, and hydrochlorothiazide may need to be decreased.

Moreover, the *V*_d_ can be influenced by drug’s protein binding. The main binding proteins are albumin, α_1_-acid glycoprotein, and lipoproteins. It has been found that albumin concentration does not change in obesity [24]; hence, drugs that mainly bind to this protein (e.g., phenytoin) do not show any changes in protein binding that require dose adjustments. However, cytokines and therefore α_1_-acid glycoprotein, which increases during inflammation, are elevated in obesity. Therefore, drugs binding to this protein (e.g., propranolol, clindamycin) could present changes to their distribution profile [19]. As a result, the dose of these drugs may need to be adjusted to achieve the desired therapeutic effect.

### Metabolism

Obesity is a disease which causes low-grade inflammation to the body tissues and decreased activity of CYP enzymes [25]. However, after bariatric surgery, liver size and intrahepatic fat decrease; therefore, this may cause an opposite result to the drugs’ metabolism and clearance, depending on their extraction rate [4•, 26]. RYGB bypasses the part of the intestine which houses a variety of metabolizing enzymes, like CYP3A4 enzymes; thus, drugs that undergo first-pass metabolism may be affected [4•]. CYP450 are the most common metabolizing enzymes, found in the liver, duodenum, and proximal jejunum. About half of the drugs in the market are metabolized by CYP3A4 (e.g., verapamil, diltiazem, nifedipine, ritonavir, erythromycin, midazolam, haloperidol); thus, bariatric procedures like RYGB, which change the anatomy of the GI tract, are expected to affect the oral bioavailability of CYP3A4 substrates. Enzymes that may also be affected include CYP2D6, CYP2C9, CYP2C19, and UDP-glucuronosyltransferases (UGTs) [4•]. Hepatic-CYP3A4 activity, within 1 year, was found to be increased following bariatric surgery, thus being inversely correlated to the patient’s body size [27, 28]. Therefore, drugs that are metabolized by CYP3A4 may be metabolized quicker, leading to decreased plasma concentrations and potential therapeutic failure. As a result, an increase in the dose of these drugs may be needed in order to achieve the desired therapeutic levels. Similarly, the content of CYP2C19 is low in the intestine, compared to the liver; thus, the intestine does not have much contribution to the first-pass metabolism of CYP2C19 substrates (e.g., citalopram, diazepam, clopidogrel) [22]. Angeles et al. found that in RYGB, hepatic CYP2C19 activity was increased, in contrast to CYP2D6 and CYP1A2, which remained the same; thus, weight loss did not cause any changes to their activity [4•]. Weight loss could potentially increase the activity of hepatic CYP enzymes; therefore, the first-pass metabolism of drugs that are substrates for these enzymes may increase, which might require an increase in their dosages [7].

### Excretion

Renal function and renal clearance may be impaired in obesity; furthermore, fluid intake post-surgery is limited, therefore reducing the excretion and increasing the exposure of the affected drugs [5•]. Moreover, obesity may lead to renal injury and reduced glomerular filtration rate (GFR) [5•]; hence, it may have implications on drug elimination rate. A simulation study by Brill et al. showed that after bariatric surgery, the clearance of low extraction ratio CYP3A substrates (e.g., cyclosporine, alprazolam, and triazolam), i.e., the fraction of drug that is removed from the blood or plasma as it crosses the eliminating organ, increased by at least 1.3 times [29]. However, no conclusions can be made for high CYP3A extraction drugs, as the intrahepatic blood flow post-surgery varies and is affected by weight loss [29].

Furosemide, which belongs to loop diuretics, is mainly absorbed in the stomach and is bound 95% to plasma proteins. When excreted, approximately half of it is eliminated in its original form through urine, while the other half is metabolized into glucuronide by the kidneys [30]. Tandra et al. studied the pharmacokinetics of furosemide after RYGB using urine and blood samples. They showed that patients who underwent RYGB had significantly shorter *t*_max_, i.e., the time that furosemide takes to reach its maximum concentration in the bloodstream, without any other significant changes [31]. Therefore, in this case, the total amount of drug absorbed and eliminated from the body may not be significantly different compared to healthy individuals.

## Pharmacokinetic Changes of Commonly Used Drugs After Bariatric Surgery (Table 1)

**Table 1:** Effects of bariatric surgery on commonly prescribed drug categories [89, 90, 55]

Drug category	Bariatric surgery’s potential effect	Recommendation based on current evidence
Antidiabetics	Post-surgery, insulin requirements drop dramatically and quickly; therefore, there is a need for reassessment of antidiabetic medications	Closely monitor diabetes medications post-surgery. Avoid oral formulations that increase hypoglycemia risk (e.g., sulfonylureas, meglitinides), as there is a rise in the hypoglycemia incidents 1 year post-RYGB. Post-surgery, as weight loss progresses, the number of antidiabetic agents and the dose will need to be reduced.
Antihypertensives	Doses will often need to be reassessed before and after bariatric surgery	Monitor closely blood pressure and frequent follow-up. Patients with diabetes should preferably continue an ACEi or an ARB, due to their protective actions in the kidneys. Diuretics should be used with caution, as along with while the surgery patients may experience dehydration.
Lipid-lowering agents	Variable and incomplete evidence exists on lipid-lowering agents	Lipid levels might decrease with weight loss, therefore close monitoring is advised every 3 months until weight loss stabilizes, and thus, if needed, lipid-lowering agents should be discontinued.
Anticoagulation and antiplatelet therapy	DOACs are absorbed in the first part of the GI tract; thus, bariatric surgery may alter their absorption Warfarin is absorbed in the proximal intestine; therefore, bariatric surgery could have an impact on its absorption Antiplatelet agents, e.g., ASA, clopidogrel, prasugrel, and ticagrelor, might increase the risk of GI bleeding; thus, the need for their administration should be reassessed	As conflicting evidence exists for DOACs, they should be used cautiously, and close drug monitoring is required. Warfarin’s dose should be initially decreased, and the INR should be monitored closely. The need for their administration should be reassessed.
Antidepressant and antianxiety drugs	Depression and anxiety are commonly seen in patients with obesity Bioavailability of antidepressants may be reduced after RYGB A study showed that SSRIs, i.e., sertraline, citalopram, and escitalopram, showed decreased AUC levels after surgery, which returned to baseline levels after 6 months	Antidepressant, antianxiety, and antipsychotic drugs need close monitoring after surgery to avoid psychiatric symptom relapse. Carefully observe patients for the psychotropics’ efficacy and any signs of toxicity. SSRI concentrations might initially decrease after surgery; however, a rebound is seen within some months; thus, close monitoring is necessary. Reassess the need for antidepressants as weight loss progresses. Lithium should be closely monitored due to the increased risk of lithium toxicity.
NSAIDs and opioids	After bariatric surgery, there is increased risk of bleeding and ulcers Slow-release analgesics are less absorbed Weight loss leads to less need for analgesia	NSAIDS should be avoided due to the increased risk of gastric ulcers and bleeding. Patients on extended-release drugs should be changed to immediate-release formulations or to different routes of administration. Close monitoring for opioid withdrawal.
PPIs	Omeprazole was found to have a significantly lower systemic exposure post-surgery, a shorter *t*_max_, and a higher *C*_max_	Rapid dissolve tablets or capsules that can be opened in accordance with the SmPC may display better absorption post-bariatric surgery.
Contraceptive pills	Reproductive-aged women are recommended to avoid pregnancy for 12–24 months after bariatric surgery, as this allows time for the body to recover from the reduced nutrient absorption	Non-oral options should be advised in order to increase their efficacy, due to the risk of malabsorption.
Antiretrovirals	Changes in the gut anatomy and function may lead to suboptimal treatment outcomes of ART	Most nucleoside analog reverse transcriptase inhibitors, darunavir and dolutegravir, are safe post-bariatric surgery. Rilpivirine should be avoided.

### Antidiabetic Medications

Diabetes is a disease that should be closely and frequently monitored post-bariatric surgery. Up until the patient’s weight stabilizes, insulin requirements may change, especially during the first months after the procedure; i.e. insulin sensitivity and secretion improve; thus, blood glucose levels decrease [32, 33].

Considering antidiabetic medications, it is advised to avoid oral formulations that increase the risk of hypoglycemia (e.g., sulfonylureas, meglitinides), as there is a rise in the hypoglycemia incidents 1 year post-RYGB [33, 34]. Post-surgery, as weight loss progresses, the number of antidiabetic agents and their dose will need to be reduced, due to the decline in blood glucose levels [35, 36]. Hemoglobin A1C (HbA_1c_) should be checked 3 months after the surgery and periodically thereafter as indicated.

Currently, there are no specific guidelines for the dose adjustments of metformin, SGLT2 inhibitors, and GLP-1 agonists. However, there is an active prospective randomized controlled trial, the BY-PLUS study, which will recruit 150 patients with obesity and T2DM, who will undergo bariatric surgery, examining the medication following bariatric surgery for T2DM [37]. Lorico and Colton suggest that if the patient is taking more than one oral medication and the HbA1c is < 9%, then metformin is the preferred single-agent therapy [7]. If HbA1c is ≥ 9%, a second agent should be given with metformin, to reduce cardiovascular risk [7].

Although a considerable number of patients may require fewer or no antidiabetic drugs within the first year after surgery, it is important to conduct regular screenings for hyperglycemia and make necessary adjustments to diabetes medications in the years post-surgery.

### Lipid-Lowering Agents

Atorvastatin is a lipid-lowering agent that has been studied after bariatric surgery. Its main metabolizing enzymes include CYP3A4 and CYP3A5, and its disposition is also affected by P-gp transporters found in the liver [14]. A study showed that 3 to 6 weeks post-RYGB, there was high interpatient variability in its systemic exposure, ranging from a threefold increase to a twofold decrease, therefore suggesting the use of the lowest effective dose [14]. Another study by Jakobsen et al. showed that the systemic exposure of atorvastatin changed significantly over time after RYGB and duodenal switch; thus, patients should be closely monitored for therapeutic effects and adverse events during the first years after bariatric surgery [38].

Gesquiere et al. studied drug disposition before and after gastric bypass of fenofibrate [39], which presents high permeability and low solubility [4•]. The results showed that the disposition of fenofibrate remained unchanged after RYGB.

However, it should be considered that lipid levels might decrease with progressive weight loss; therefore, monitoring is important every 3 months until weight loss stabilizes and, if needed, lipid-lowering agents should be discontinued [7].

### Anticoagulation and Antiplatelet Therapy

Direct oral anticoagulants (DOACs) are absorbed in the first part of the GI tract; thus, bariatric surgery which causes changes in the absorptive surface could alter their absorption [40]. Rivaroxaban, a factor Xa inhibitor, is metabolized by many CYP enzymes, including CYP3A4 and CYP2J2. It has a high oral bioavailability (80–100%) and low degree of first-pass metabolism [41]. Since it has multiple elimination pathways, i.e., renal and metabolic degradation, it has been found to present similar systemic exposure pre- and post-surgery, independent of the type of procedure [42]. A study found that RYGB and SG did not seem to alter its pharmacokinetics up to 8 months following surgery [43]. However, another study concluded that DOACs, especially rivaroxaban, should be used cautiously after bariatric surgery, due to significantly lower peak concentrations post-surgery [44].

Other studies have shown that rivaroxaban and apixaban do not require dose adjustment after bariatric surgery [7, 45, 46]. Therefore, as conflicting evidence exists for DOACs, they should be used cautiously, and drug monitoring is required. Regular review appointments are needed to assess for signs of bleeding or anemia, adverse effects, and features of thromboembolic events. Several methods exist for assessing hemostasis while on DOACs, such as obtaining a baseline activated partial thromboplastin time (aPTT), prothrombin time (PT), or both. However, these tests provide only qualitative data, as they lack sensitivity and do not provide a correlation between dosage and response. They are used to establish whether on-therapy or toxicity levels are present [47]. To ensure appropriate dosing of factor Xa inhibitors, it is recommended to measure drug levels 2–4 h after the morning dose, using a chromogenic anti-Xa assay specific to the drug. This should be done 3–5 days after initiating the treatment while the results should be compared against the expected therapeutic ranges. If the drug level falls within the expected range, the treatment should continue, and a second drug level should be measured 4–6 months post-surgery [48]. Patients should be informed that the optimal choice of oral anticoagulant after bariatric surgery is uncertain and that they should be involved in shared decision-making to compare the proposed strategy with prescribing a vitamin K antagonist [48].

Warfarin, a vitamin K antagonist, which is metabolized primarily by CYP1A2 and CYP3A4 [49], is absorbed in the proximal intestine; therefore, bariatric surgery could have an impact on its absorption [8•]. Studies have showed that warfarin dose had to be significantly reduced 6 months postoperatively and 6 months to 1 year after the surgery, its levels gradually went back up to pre-surgical levels [50, 51]. In order to decrease the risk for bleeding, warfarin’s dose should be initially decreased, while the international normalized ratio is closely monitored [7]. Moreover, it is very important to counsel patients that an increased amount of dietary intake of vitamin K may reduce the efficacy of warfarin. Vitamin K–rich foods include broccoli, asparagus, spinach, and kale. Therefore, patients should be advised to eat the same amount of vitamin K every day [52].

It is known that antiplatelet agents, such as acetylsalicylic acid (ASA), clopidogrel, prasugrel, and ticagrelor, might increase the risk of GI bleeding; thus, the need for their administration should be reassessed; however, studies have shown different results. For patients with high risk of cardiovascular events, the lowest effective dose should be prescribed [7, 17]. ASA is absorbed in the stomach as an unionized form and in the duodenum as partly ionized, and its rate and extent of absorption increase after RYGB [39]. This suggests that the absorption of the ionized form can also take place in the jejunum, due to the increased gastric emptying time [4•, 22]. However, while the AUC and *C*_max_ are increased, this was not clinically important for patients’ response [4•]. A study by Mitrov‐Winkelmolen et al. has shown that even though the *C*_max_ and AUC_0–24 h_ of ASA were higher after surgery, the dosing range for the inhibition of platelet aggregation was within the recommended range. Therefore, there are no clinically relevant changes in the pharmacokinetics of ASA after surgery and no dose adjustment is recommended [22]. Moreover, another study showed that low-dose ASA did not increase the risk of bleeding after RYGB [53]. Ma and Norgard found that bariatric surgery improved the pharmacodynamic response of ticagrelor that was blunted by obesity [54]. In conclusion, the aforementioned studies have shown that there is no need for dose adjustments of platelet inhibitors after bariatric surgery [8•].

### Antihypertensive Drugs

After bariatric surgery, the need for antihypertensive drugs should be reassessed, blood pressure should be closely monitored, and frequent follow-up is highly recommended [7]. Concerning the study of oral metoprolol, a cardioselective beta-1-adrenergic receptor inhibitor that is mainly metabolized by CYP2D6, Gesquiere et al. showed that RYGB did not have any significant effects in its systemic exposure 6 and 8 months after surgery [39]. It is advised to monitor blood pressure and adjust the dose accordingly if needed.

The available literature is limited with regard to the pharmacokinetics of beta-blockers after bariatric surgery. Although atenolol and propranolol are alkaline, propranolol is lipophilic while atenolol is hydrophilic. Restrictive bariatric surgeries, which decrease secretion of hydrochloric acid and result in less acidic conditions in the stomach, favor the absorption of basic drugs, including beta-blockers [55]. However, the increased stomach pH reduces the solubility of alkaline drugs in the stomach, which improves their absorption in the intestine [55]. Hence, in cases where it is essential to use beta-blockers after a bariatric procedure, it is better to prescribe a hydrophilic agent like atenolol [56].

A study by Yska et al. showed that RYGB surgery can affect the bioavailability of metoprolol from an immediate-release tablet, and thus, it is important to closely monitor patients and adjust the dose if necessary. Moreover, RYGB significantly decreases the bioavailability of metoprolol from a controlled release tablet, and healthcare professionals may need to increase the dose based on the patient’s clinical response [57].

On the other hand, patients with diabetes that need antihypertensives should preferably continue an angiotensin-converting enzyme (ACE) inhibitor or an angiotensin II receptor blocker (ARB), due to their protective action in the kidneys [7]. Moreover, angiotensin is overexpressed in obesity, which may lead to obesity-related hypertension. Therefore, ACE inhibitors and ARBs are ideal for the management of hypertension in patients with obesity [58]. However, there are some considerations to take into account. Enalapril, an ACE inhibitor, is a prodrug, whose effectiveness might be decreased, because it transforms into the active form in the stomach; therefore, it may be necessary to switch to a different ACE inhibitor [59]. Furthermore, the absorption of ramipril, another ACE inhibitor, may be reduced in patients who have steatorrhea; thus, its effectiveness should be assessed and another option should be considered if necessary [59].

As far as diuretics are concerned, they should be used with caution, while surgery patients may experience dehydration, thus exacerbating their effect.

### Antidepressant and Antianxiety Drugs

Depression and anxiety are commonly seen in patients with obesity, especially in women [60]; furthermore, these patients often use psychotropic medications. Moreover, despite drastic weight loss, some psychological problems may be present after the bariatric procedure, which need to be investigated. However, healthcare professionals should be aware of their potential decreased serum concentrations after bariatric procedures, which can develop even in the first weeks.

Venlafaxine, a serotonin-norepinephrine reuptake inhibitor (SNRI), metabolized by CYP2D6, did not show any significant difference exposure or absorption after RYGB [61]. On the other hand, duloxetine, another SNRI metabolized by CYP2D6, and sertraline, a selective serotonin reuptake inhibitor (SSRI), were shown to have a decreased exposure after RYGB, which may last at least 12 months post-surgery; thus, an increase in dosage should be considered if the patient is undertreated [62]. Moreover, a study found that SSRIs, i.e., sertraline, citalopram, and escitalopram, showed decreased AUC levels after surgery, which returned to baseline levels after 6 months [63].

Lithium, which is a mood stabilizer and a drug with a narrow therapeutic index, should be closely monitored due to the increased risk of lithium toxicity. Lithium and bupropion, a norepinephrine-dopamine reuptake inhibitor (NDRI), were found to present a faster dissolution after RYGB in an in vitro study [64].

Regarding all antidepressant, antianxiety, and antipsychotic drugs, close monitoring after surgery is advised in order to avoid psychiatric symptom relapse. Patients should be carefully observed for the psychotropics’ efficacy and any signs of toxicity. Studies have shown that SSRI concentrations might initially decrease after surgery; however, a rebound effect is seen within some months; thus, strict monitoring is necessary [63, 65].

### Non-steroidal Anti-inflammatory Drugs (NSAIDs) and Opioids

After bariatric surgery, some common complications include bleeding and ulcers. Therefore, the use of NSAIDS should be avoided, due to the increased risk of gastric ulcers and bleeding [18], which may develop 1 year after surgery [8•]. To date, there are no studies on the pharmacokinetics of NSAIDS before and after bariatric surgery; thus, their use should be discouraged [8•].

Several studies have suggested that while overall medication for pain control decreases considerably after bariatric surgery, the use of opioids not only persists but also rises for many patients [66]. There is a lack of clarity regarding the use of opioids after bariatric surgery, and there are not many guidelines available to guide healthcare providers [67]. A retrospective cohort study has shown that bariatric surgery was associated with a greater risk of chronic prescription opioid use (CPOU) incidence compared to non-surgical controls [68].

Morphine is mainly metabolized in the liver by UGT2B7, which belongs to the UDP-glucuronosyltransferases. A study showed that following RYGB, its clearance was related to the patient’s BMI. Moreover, 6 months postoperatively, its rate of absorption was significantly higher, whereas the *t*_max_ decreased and *C*_max_ increased, due to the faster gastric emptying [69].

Moreover, patients on extended-release drugs or high-dose opioids, that will undergo malabsorptive procedures, should be changed to immediate-release formulations or to different routes of administration [7]. Limiting the prescribed opioid to the lowest effective dose for the shortest effective duration is advised, as well as regular monitoring and reassessment of opioid need.

### Proton Pump Inhibitors (PPIs)

As noted earlier, after bariatric surgery, gastric ulcers may develop; thus, there is a common need for PPIs as prophylactic use. Omeprazole, a PPI, primarily metabolized by CYP2C19, was found to have a significantly lower systemic exposure post-surgery, a shorter *t*_max_, and a higher *C*_max_ due to the increased activity of CYP2C19 [39]. Collares-Pelizaro et al. found that patients who have undergone bariatric surgery are unable to effectively block the production of hydrochloric acid, which can lead to the formation of peptic injuries, as the standard dosage of omeprazole, i.e., 40 mg, given after the procedure is insufficient to achieve the necessary serum levels [70].

However, a study by Portolés-Pérez et al. showed that even if patients with obesity who undergo RYGB experience a decrease in the absorption of omeprazole 1–6 months after the procedure, its pharmacokinetic parameters are comparable to those in control subjects 6 months after RYGB. Therefore, there is no need to adjust the dosage of omeprazole after RYGB [71]. Consequently, conflicting results exist concerning the dosage of PPIs after bariatric surgery; thus, there is a need for more research.

Schulman et al. reported that soluble PPIs, i.e., open capsules, might be easier absorbed than intact capsules [72]. Thus, rapid dissolve tablets or capsules that can be opened in accordance with the summary of product characteristics (SmPC) of the product may display better absorption post-bariatric surgery [7, 8•].

### Contraceptive Pills

According to the American and European guidelines, women of reproductive age are recommended to avoid pregnancy for 12–24 months after bariatric surgery [8•]. There is a lack of substantial evidence on the safe and effective use of contraception after bariatric surgery, as no randomized controlled trials or long-term observational studies have been carried out. There are concerns that postoperative complications like long-term diarrhea or vomiting could potentially decrease the effectiveness of oral contraceptives.

In a small study by Ginstman et al., oral desogestrel was not found to present any changes in the absorption rate or its systemic exposure 4 and 12 months post-RYGB [73]. Moreover, Ciangura et al. showed reduced levels of norgestrel 6 months post-RYGB, but still high enough levels in order to produce sufficient contraception in women [74].

However, after bariatric surgery, other studies [75, 76] have recommended that oral contraceptive should not be considered due to the decreased absorption area and enterohepatic circulation, which may lead to suboptimal efficacy after malabsorptive bariatric procedures. Due to increased risk of chronic diarrhea following malabsorptive and restrictive procedures, non-oral options should be advised in order to increase the efficacy of contraceptive drugs, such as implants [8•]. Even if some studies showed no differences in the pharmacokinetics of oral contraceptives after bariatric procedures, clinical outcomes should be evaluated. Damhof et al. showed that 16% of women use unsafe contraception methods after bariatric surgery [77]; thus, contraceptive and pregnancy counseling is of paramount importance. Therefore, it is suggested to avoid using oral contraception after malabsorptive and restrictive-malabsorptive procedures, as the major estrogenic component of oral contraceptives, 17α-ethinyloestradiol (EE2), undergoes first-pass metabolism [78].

According to the Faculty of Sexual & Reproductive Healthcare (FSRH) clinical guideline, the available data on the effectiveness of oral contraceptives in women who have undergone bariatric surgery is very limited and often contradictory [79]. Thus, it is difficult to provide a clear recommendation on the use of oral contraceptives after bariatric surgery. Women who have undergone surgery should be informed that oral contraceptives may be less effective, and they should consider using alternative methods of contraception instead. Non-oral options may be a better choice [79].

### Transplant Medications

Chan et al. studied immunosuppressive medications after laparoscopic SG, i.e., tacrolimus, extended‐release tacrolimus, mycophenolate mofetil, and enteric‐coated mycophenolate sodium [80]. The AUC_0–24 h_ of the drugs increased after the surgery, while the total clearance was decreased for tacrolimus, mycophenolate mofetil, and enteric‐coated mycophenolate sodium. SG may be associated with significant changes in pharmacokinetics of immunosuppressive [80]. Therefore, it is important to monitor the level of immunosuppression to target the desired effect and avoid drug toxicity.

### Antibiotics

Obesity increases the risk for infections, including surgical wound and skin infections. The effects of various antibiotic categories, such as beta lactams, macrolides, and fluoroquinolones, have been studied after bariatric surgery.

Rocha et al. and Montanha et al. studied the effects of oral amoxicillin after RYGB [81, 82]. The first study found a significant rise in the AUC and *C*_max_ of the antibiotic post-surgery, whereas the second study found a higher AUC for amoxicillin suspension *vs.* amoxicillin tablets. However, oral amoxicillin may be used post-RYGB surgery, as the time above the minimum inhibitory concentration (MIC) for pathogens with a MIC < 4 mg/L was attained.

Two more studies evaluated the effects of macrolides, i.e., azithromycin and erythromycin, post-surgery and found a reduction in exposure after surgery [83, 84]. The authors suggested that the use of macrolides should be questioned after bariatric surgery, because of potential early treatment failure, the potential need for dose modification, and close monitoring. Azithromycin AUC was reduced by 30% in gastric bypass subjects compared with controls. The potential for early treatment failure exists, and dose modification and/or closer clinical monitoring of gastric bypass patients receiving azithromycin should be considered.

Fluoroquinolones, i.e., ciprofloxacin and moxifloxacin, have been studied by De Smet et al. and by Rivas et al. The first study showed that oral and intravenous exposures of moxifloxacin in patients who have undergone RYGB were 50% higher than in patients without RYGB, due to a higher enterohepatic recirculation of the drug after gastric bypass [85]. The second one showed that the AUC of ciprofloxacin decreased after the surgery, but it returned to normal values 6 months after RYGB; thus, it is not necessary to modify the doses of ciprofloxacin in these patients [86].

### Midazolam

Brill et al. did two studies for the effects of midazolam, metabolized by CYP3A, after bariatric surgery. The first study showed that after RYGB and SG, hepatic intrinsic clearance increased 1.7-fold, due to the increased hepatic CYP3A4 activity, but its oral bioavailability did not change, which was explained as an increase in fraction escaping intestinal first-pass metabolism [27, 29]. The second study showed that 3 and 12 months postoperatively, the rate of oral midazolam’s absorption was faster [27, 29].

### Antiretroviral Therapy

Patients living with human immunodeficiency virus (HIV) must always maintain sufficient antiretroviral exposure and activity to prevent the development of resistance and disease progression [87]. Pharmacokinetics of antiretroviral therapy (ART) for patients with HIV may be affected after bariatric surgery, as changes in gut anatomy and function may lead to suboptimal treatment outcomes of ART.

There have been some concerns regarding the absorption of oral highly active antiretroviral therapy (HAART), as some studies suggest a drop in their serum concentration [88]. However, CD4 count and viral load do not appear to be impacted. Nausea, vomiting, and dysphagia, which may occur after bariatric procedures, may lead to an inability to administer HAART. Thus, liquid or parenteral formulations may be suggested [88].

Zino et al. suggest that most nucleoside analog reverse transcriptase inhibitors; the protease inhibitor, darunavir; and the integrase strand transfer inhibitor (INSTI), dolutegravir, are successful drug candidates after bariatric surgery [87]. In contrast, atazanavir has a risk of viral failure due to the risk for lower absorption. However, few data are available for non-nucleoside reverse transcriptase inhibitors after bariatric surgery. Due to its unfavorable pharmacokinetics, rilpivirine should be avoided. Even though doravirine shows favorable drug characteristics, clinical studies after bariatric surgery are not existing in order to make any conclusions [87].

Currently, there are no specific guidelines regarding the management of HIV-infected patients after bariatric surgery. Published studies on patients with HIV who have undergone bariatric surgery are limited to case reports; thus, prospective clinical trials are needed in order to make robust conclusions. Healthcare providers must monitor ART regimens and adjust the doses accordingly.

## Conclusions

Bariatric surgery is increasingly used for patients with severe obesity as it has been proven to lower long-term morbidity and mortality. However, significant weight loss may cause changes in the pharmacokinetics of drugs. Unfortunately, limited data are available, and the lack of existing guidelines frequently leads patients to experience either drug toxicity or therapeutic undertreatment after bariatric surgery. Pharmacokinetic parameters to be taken into consideration postoperatively include gastric motility, gastric volume and pH, surface area, bile secretions, carrier proteins, and first-pass metabolism [7]. Factors to be monitored closely include plasma drug levels, patients’ clinical outcomes, and laboratory markers, especially for drugs with a narrow therapeutic index. Until more evidence emerges, patients should be followed up frequently and treated in accordance with their response to the drug therapy [7].

## Data Availability

The data that support the findings of this study are available from the corresponding author, upon reasonable request.
